# Height and Light-Obtaining Ability of *Leymus chinensis* Increased After a Decade of Warming in the Typical Steppe of Inner Mongolia, China

**DOI:** 10.3390/plants14111702

**Published:** 2025-06-03

**Authors:** Zhiqiang Wan, Rui Gu, Yan Liang, Xi Chun, Haijun Zhou, Weiqing Zhang

**Affiliations:** 1 Key Laboratory of Mongolian Plateau’s Climate System of Inner Mongolia Autonomous Region, Inner Mongolia Normal University, Hohhot 010020, China; wanzhiqiang@imnu.edu.cn (Z.W.); chunxi@imnu.edu.cn (X.C.); hjzhou@imnu.edu.cn (H.Z.); 20180036@imnu.edu.cn (W.Z.); 2 College of Geographical Science, Inner Mongolia Normal University, Hohhot 010020, China; 3 College of Grassland and Resources Environment, Inner Mongolia Agricultural University, Hohhot 010022, China; 4 Ordos Forestry and Grassland Science Institute, Ordos 017010, China; abcd20010101@126.com

**Keywords:** *Leymus chinensis*, photosynthetic efficiency, resource utilization, ability to obtain light, leaf anatomical structure

## Abstract

In the era of global climate change, existing evidence indicates that dominant species play a crucial role in regulating grassland structure and function. However, what remains overlooked are the factors that regulate the growth of dominant species under climate change. Some studies have indicated that the future climate of the Inner Mongolia grasslands will specifically show a trend of warming and humidification. Hence, in 2013, we conducted a controlled warming and precipitation addition experiment in a temperate steppe in Inner Mongolia, China. Open-top chambers (OTCs) were used to simulate warming (by 1.5 °C) and rainfall (twice a month, 10% of the average precipitation between 1960 and 2011 of the same month each time) during the growing season. In 2023, the resource utilization efficiency, morphological characteristics, leaf anatomical structure, and population quantity characteristics of the dominant species (*Leymus chinensis*), and community species diversity were monitored under control (CK), warming (T), precipitation addition (P), and warming plus precipitation addition (TP) conditions. We found that the plant height of *L. chinensis* significantly increased under warming; its height was 41.97 cm under TP, 41.84 cm under T, 29.48 cm under P, and 28.88 cm under CK. We observed that *L. chinensis* primarily obtains more light by increasing leaf area and height under warming conditions. Environmental changes also alter the tissue structure of *L. chinensis* leaves, leading to a decrease in lignification after increasing the water content. In this study, warming significantly increased the *L. chinensis* leaf area but decreased the leaf carbon content. Warming and precipitation addition regulated the height of *L. chinensis* by affecting the leaf area, leaf–stem ratio, and distance of the bottom leaf from the ground. Our results provide reasonable predictions regarding the succession direction of the *L. chinensis* steppe under global climate change in the future.

## 1. Introduction

The responses of a plant community’s structure and composition to global climate change are strong [1,2]. Climate change can alter a plant community’s composition and biomass production by changing species dominance, physiological processes (of individual species), and interspecific competition [3,4,5,6].

Since the Industrial Revolution, the world’s global average temperature has shown a significant upward trend, increasing by 1.1 °C from 1901 to 2022 [7], and the trend has been more rapid in the world’s high-altitude and high-latitude regions [8]. Over this period, the global precipitation pattern has simultaneously undergone drastic changes [9]. According to predictions, the average annual precipitation in mid to high latitudes around the world will increase in the future, with prominent additional changes in regional and seasonal precipitation expected by the end of the 21st century [7]. In this context, grassland ecosystems (deemed an important type of terrestrial ecosystems), which are sensitive to climate change, may be affected by the effects of climate change in the future.

An ecosystem’s dominant plant species has a higher degree of abundance compared to other species, along with significant control over plant community structure and environmental conditions. Their status and development trends within a community greatly affect the community’s stability and species diversity [6,10]. Dominant species account for the majority of an ecosystem’s resources due to their competitive edge. The loss of characteristics that enable an ecosystem’s dominant species to adapt to its average environmental conditions can profoundly impact the ecosystem’s processes [6]. An ecosystem’s dominant species exhibit a greater response to environmental disturbances than its non-dominant species [11]. Dominant species also have a greater competitive advantage than other species with regard to resisting environmental interference, which makes them more sensitive to climate change [12].

Notably, the grasslands in northern China’s semi-arid and arid regions are an important component of the Eurasian grassland terrestrial ecosystem [13]; they are also deemed the most widely distributed natural vegetation in the abovementioned regions. The dominant species in the steppe is the perennial rhizomatous *Leymus chinensis*. According to the existing vegetation map of the world, the total global area covered by *L. chinensis* grassland is estimated to be 420,000 km^2^, half of which is situated in China. This grassland is considered one of the most widely distributed formations in the temperate grassland regions of China. The largest natural *L. chinensis* grassland is situated in Inner Mongolia, China, comprising an area of about 108,700 km^2^ [14]. Owing to the sensitivity of individual traits of *L. chinensis* to changes in the larger plant community environment, it is often considered an excellent subject to study plant community dynamics [14,15,16,17,18]. High temperature and drought treatments have caused a significant decrease in the photosynthetic rate (Pn) of *L. chinensis*, while precipitation addition has significantly increased the Pn of *L. chinensis* [19]. Moreover, the chlorophyll content in *L. chinensis* leaves is closely related to the integrity of its chloroplasts and directly proportional to its photosynthetic capacity [19,20]. Furthermore, the changes in key plant traits related to resource acquisition and environmental adaptation, such as plant height, specific leaf area (SLA), leaf nitrogen content, and d^13^C, have been reported as responses to climate change [21,22,23]. Changes in precipitation serve as the main factor affecting the growth and functional traits of plant species in semi-arid grasslands [24]. Others have observed that a species’ SLA and leaf nitrogen content decrease with increasing precipitation [23]; plants may also alter their phenotypic plasticity to adapt to different water conditions [25]. In addition, some scholars have found that the height of *L. chinensis* decreases with drought, confirming a decrease in plant growth rate under drought conditions [26]. Drought during the growing season is also considered an important factor limiting plant growth in semi-arid regions [27]. A significant positive correlation between plant height and d^13^C indicates that the combination of plant height and d^13^C can reflect the adaptive strategies of plants in arid environments. Changes in d^13^C based on alterations in stomatal conductance and photosynthetic capacity can explain this relationship: drought-induced stomatal closure has been associated with an increase in d^13^C in leaf tissue [28].

Experimental research can comprehensively record the impact of climate change on grassland plants. However, the duration of such research may affect its results. In fact, ecosystems typically have a stress response to short-term climate change but may adapt to environmental changes over a longer period of time. For example, on a high-fat grassland in Oklahoma, community composition remained resistant to experimental warming for the first seven years. However, it showed slight fluctuations in species diversity in the eighth year [29]. Therefore, in order to understand the significant changes in species diversity caused by climate change, it may be necessary to consider extending the time [30]. In other words, it is necessary to conduct continuous field experiments in order to observe the impact of climate change on ecosystems. The height variation of dominant plants in a community under climate change conditions is crucial, and the importance of plant height to ecosystems has been overlooked. However, climate change promotes carbon sequestration in ecosystems by increasing plant community height. Most previous studies have focused on the impact of a single climate factor (such as temperature or precipitation), with insufficient research on the synergistic/antagonistic effects of warming and precipitation. Additionally, there is limited research on the relationship between leaf anatomical structure (such as vascular bundle characteristics) and plant height growth, which limits the understanding of functional traits in climate response strategies [21].

In this study, we conducted a controlled warming and precipitation addition experiment in Inner Mongolia, China, which started in 2013. We found that the height of *L. chinensis* increases under warming conditions. Thus, from the perspectives of photosynthetic efficiency, resource utilization, ability to obtain light, and leaf anatomical structure, our hypothesis was that warming and precipitation addition increases the photosynthetic and water use efficiencies of *L. chinensis*, thereby leading to greater growth advantages.

## 2. Materials and Methods

### 2.1. Study Site

**Figure 1:**
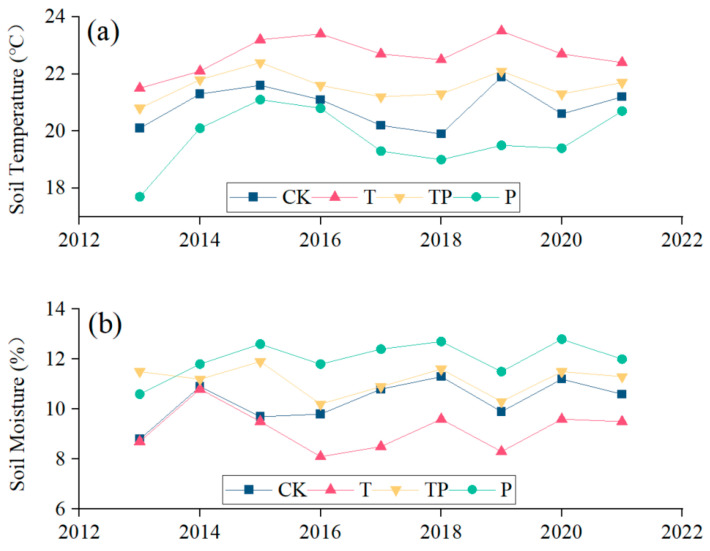
Effects of warming and precipitation addition on soil temperature and soil moisture. (**a**) Soil temperature; (**b**) soil moisture under different treatments.

Our experiment was conducted at the Grassland Ecology Research Base of Inner Mongolia University, which is located in the typical steppe of Inner Mongolia. The experiment began in July 2013, and open-top chambers (OTCs) were used for warming. Fans were installed inside to control the temperature increase range. The precipitation addition range was 20% of the average monthly precipitation from 1961 to 2010, and the precipitation addition was carried out twice in each month of the growing season. The specific experimental setup and methods were consistent with those of Wan et al., 2023 [31]. Four treatments were set up, including control (CK), warming (T), warming plus precipitation addition (TP), and precipitation addition (P), with four replicates for each treatment; these enabled us to obtain a total of 16 plots. The area of each plot was 3 m × 3 m. The area investigated in each plot was the same as the bottom area of the OTC. Notably, our experiment significantly impacted the study location’s soil temperature and soil moisture, as shown in Figure 1.

### 2.2. Data Measurements and Analysis

#### 2.2.1. Plant Height

We selected 20 individual *L. chinensis* specimens in each plot to calculate their mean height in July 2023, which is the peak growing season for grassland plants; the heights of the nutrient branches were observed in their natural state without the branches being artificially straightened.

#### 2.2.2. Resource Utilization Efficiency

Three aspects—net photosynthetic rate, transpiration rate, and stomatal conductance per unit leaf area (LA)—were used to represent the photosynthetic efficiency of *L. chinensis* leaves. Five plants—those considered healthy and well-growing, with fully expanded leaves—were chosen from each plot and measured using an open gas exchange system (LI-6400, Li-COR Inc., Lincoln, NE, USA) between 08:30 h and 11:30 h on a sunny and cloudless day from May to August in 2023 and measured twice a month, with an interval of about 15 days. Ten data points were recorded for each blade to calculate the average value for each measurement. Non-overlapping blades were unfolded and spread flat to fill the entire leaf chamber, which had an area of 2 cm × 3 cm. The CO_2_ concentration in the leaf chamber was controlled at 380 ± 20 μ mol·mol^−1^, the leaf chamber temperature was kept at 25–28 °C, the relative humidity of the air was kept at 50–70%, and the photosynthetically active radiation (PAR) provided by the red and blue light sources was 1500 μmol·m^−2^·s^−1^ [32]. From each plant, a single leaf was selected; the chlorophyll content of these leaves was measured using a handheld chlorophyll meter (SPAD-502Plus, Konica Minolta, Japan).

Thereafter, we decided to represent nutrient utilization efficiency as leaf C/N/P. To gauge the same, we selected well-grown, disease-free *L. chinensis*, collecting 5–15 complete leaves from each selected plant and drying them at 65 °C. The dried plant samples were subsequently ground using a ball mill and filtered through a 100 mesh sieve. Then, both the C and N contents were determined using an element analyzer (Vario EL Cube CHNOS Elemental Analyser, Elementar Analysensysteme GmbH, Hanau, Germany). Meanwhile, P concentrations were measured and analyzed using inductively coupled plasma mass spectrometry (5100 ICP-OES, Perkin Elmer, Waltham, MA, USA).

#### 2.2.3. Morphological Characteristics

To measure our specimens’ LA and SLA as morphological trait indicators, we selected 10 healthy plants (with uniform growth) from each plot to determine LA. The Epson V19 scanner was used to scan the images of these plants’ flattened leaves; the LA of each leaf was measured using ImageJ 1.53e (LOCI, University of Wisconsin, Madison, WI, USA). All the leaves were subsequently placed in envelopes, oven-dried at 65 °C, and weighed. Then, the following definitions were outlined: SLA = LA/dry leaf weight; ratio of leaf to stem = dry leaf weight/dry stem weight.

We also measured the bottom leaf height (from ground level) of 10 *L. chinensis* specimens using a meter ruler; this represented the ability of *L. chinensis* to obtain light.

#### 2.2.4. Leaf Anatomical Structure

Three fresh *L. chinensis* leaf tissue samples were collected from each plot in July 2023 and fixed with FAA (formaldehyde: glacial acetic acid: ethanol = 0.5:0.5:9) for a timespan exceeding 24 h. The selected leaves were dehydrated, soaked in wax, embedded, and sliced. Then, dewaxing and staining were performed on these leaves with safranin–alcian green, and subsequently, they were sealed with neutral gum to render the dewaxing and staining permanent. Later, we observed the anatomical structure of the leaves using a 100× optical microscope and ensured that all blade structures were observed using the same magnification.

#### 2.2.5. Data Analysis

Before conducting our analysis, we checked the normality of distributions and homogeneity of variance pertaining to our data using the Shapiro–Wilk and Bartlett tests. Excel 2020 was used for preliminary data arrangement, while multivariate ANOVA was used to illustrate the significance of relevant indices under different treatments (as per SPSS 21.0; SPSS Inc., Chicago, IL, USA). Moreover, linear regression analysis was conducted using SPSS 21.0 (SPSS Inc., Chicago, IL, USA). Furthermore, factor analysis was performed using Origin Pro 2021 (OriginLab, Northampton, MA, USA). AMOS 21.0 (Amos Development Co., Armonk, NY, USA) was additionally used to analyze the relationship between different variables with the aid of a structural equation model (SEM). Notably, for our data analysis, we selected the best model with the lowest Akaike Information Criterion value and a chi-square value (χ2) > 0.05, a value of *p* > 0.05, and a value of RMSEA < 0.05.

## 3. Results

### 3.1. Height of L. chinensis

**Figure 2:**
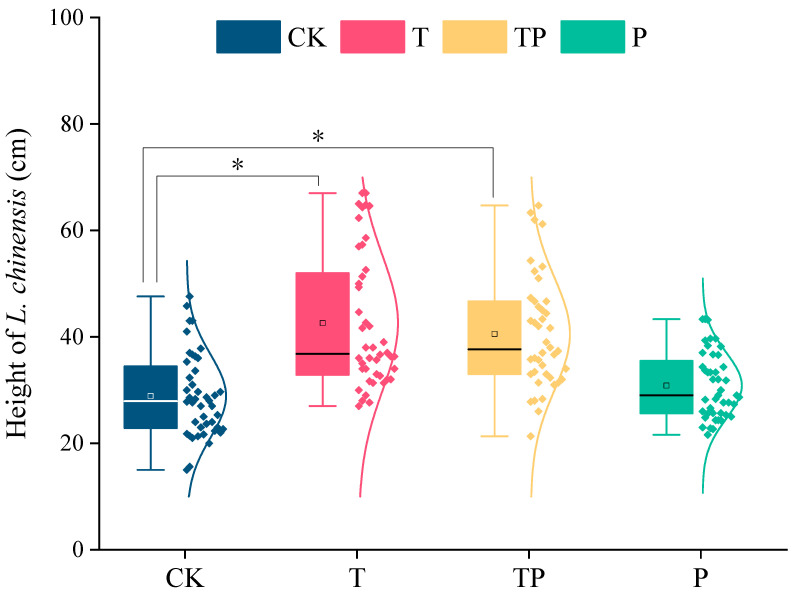
Effects of warming and precipitation addition on density, productivity, and height of *L. chinensis*. CK: control, without warming and precipitation addition; T: warming; P: precipitation addition; TP: warming plus precipitation addition; * significant at *p* < 0.05. The box represents the interquartile range (IQR), encompassing the middle 50% of the data. The horizontal line within the box indicates the median value. Whiskers extending vertically from the box delineate the full data range from minimum to maximum values. Individual data points are displayed as distinct markers, each corresponding to an experimental measurement.

In this study, the height of *L. chinensis* was 41.97 cm under TP, 41.84 cm under T, 29.48 cm under P, and 28.88 cm under CK. Compared to CK, T and TP increased the height of *L. chinensis* by 45% (Figure 2). Warming significantly increased the height of *L. chinensis*, but precipitation addition had no significant effect on height.

### 3.2. Photosynthetic Capacity of L. chinensis

**Figure 3:**
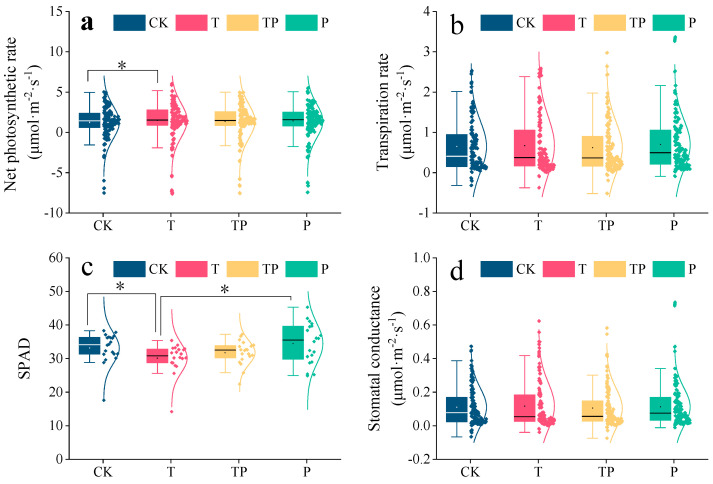
Effects of warming and precipitation addition on photosynthetic physiological capacity of *L. chinensis*. CK: control, without warming and precipitation addition; T: warming; P: precipitation addition; TP: warming plus precipitation addition; * significant at *p* < 0.05. (**a**) Net photosynthetic rate, (**b**) transpiration rate, (**c**) leaf chlorophyll content, and (**d**) stomatal conductance under four treatments. The data in (**a**–**d**) represent the values of *L. chinensis* under different treatments. The box represents the interquartile range (IQR), encompassing the middle 50% of the data. The horizontal line within the box indicates the median value. Whiskers extending vertically from the box delineate the full data range from minimum to maximum values. Individual data points are displayed as distinct markers, each corresponding to an experimental measurement.

The *L. chinensis* photosynthetic rate significantly increased under warming as compared with the rate under control conditions: by 1.49 μmol/m^2^/s under T and by 1.24 μmol/m^2^/s under CK (a 20% increase) (*p* < 0.05). Meanwhile, P exhibited a significantly increased leaf chlorophyll content compared to T (*p* < 0.05); SPAD (Soil Plant Analysis Development) was 34.49 under P and 30.13 under T (Figure 3). Warming significantly increased the net photosynthetic rate of *Leymus chinensis*, but was not conducive to leaf chlorophyll content, indicating that the increase in photosynthetic rate of *Leymus chinensis* under warming conditions is influenced by other factors.

### 3.3. L. chinensis: Ability to Obtain Light

**Figure 4:**
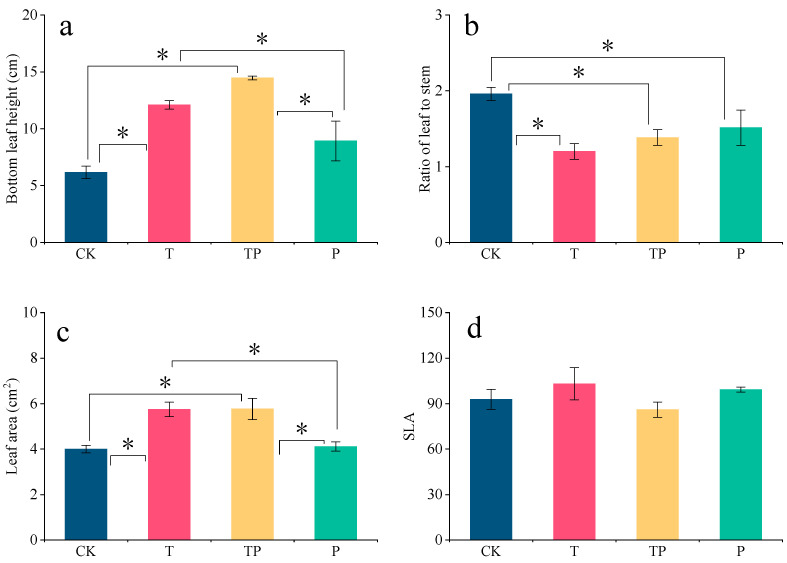
Effects of warming and precipitation addition on leaf traits of *L. chinensis*. CK: control, without warming and precipitation addition; T: warming; P: precipitation addition; TP: warming plus precipitation addition; * significant at *p* < 0.05. (**a**) Bottom leaf height, (**b**) ratio of leaf to stem, (**c**) leaf area, (**d**) SLA under four treatments. The data in (**a**–**d**) represent the mean ± SD of *L. chinensis* under different treatments.

At the outset, the selected specimens’ bottom leaf heights were 6.17 cm under CK, 12.1 cm under T, 14.47 cm under TP, and 8.93 cm under P. Warming caused the leaves of *L. chinensis* to distance themselves further from the ground, and their higher heights eventually enabled them to receive more light; this was also why *L. chinensis* grew taller under warming conditions, consistent with the Red Queen effect (any evolutionary improvement of a species may constitute evolutionary pressure on other species, and interspecies relationships may drive population evolution when environmental conditions are relatively stable). Moreover, the selected specimens’ LAs were 4.00 cm^2^ under CK, 5.75 cm^2^ under T, 5.77 cm^2^ under TP, and 4.11 cm^2^ under P; warming conditions increased LA by 44% compared to CK (Figure 4).

### 3.4. Resource Utilization Efficiency of Leymus Chinensis

**Figure 5:**
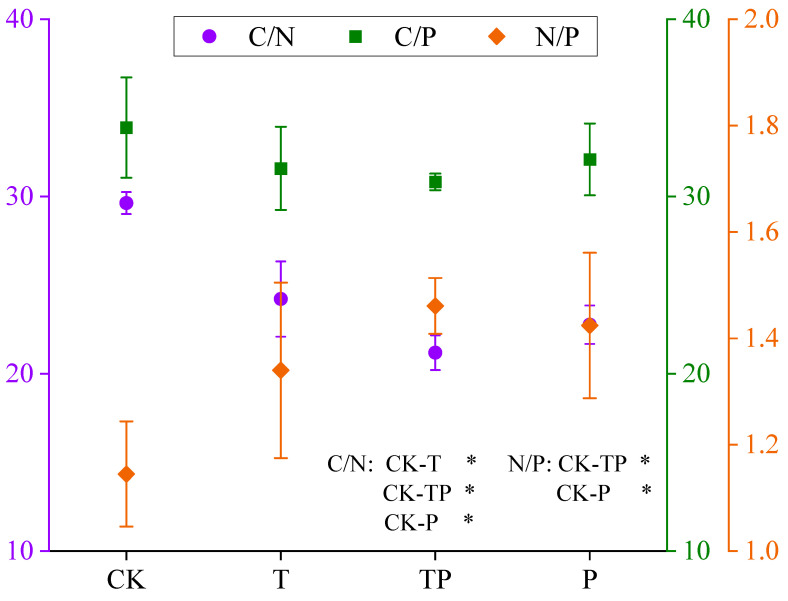
Effects of warming and precipitation addition on C/N/P of *L. chinensis*. CK: control, without warming and precipitation addition; T: warming; P: precipitation addition; TP: warming plus precipitation addition; * significant at *p* < 0.05.

The C/N of the leaves was 29.63 under CK, 22.77 under P, 24.22 under T, and 21.19 under TP. Notably, the C/N of the leaves decreased, whereas the nitrogen utilization efficiency of the plants increased after warming and precipitation addition (Figure 5). During plant growth, the C:N ratio in the leaves represents the photosynthetic fixation capacity of C through N accumulation, while the N:P ratio in the leaves indicates plant nutrient limitations in unfavorable habitats. The leaf nitrogen–phosphorus ratio can be used to indicate vegetation composition and nutrient limitations at the community level. Warming and precipitation addition reduced the ability of *L. chinensis* to fix carbon through N, but precipitation addition increased the N/P of *L. chinensis*, indicating that precipitation addition improved N and P utilization efficiency to compensate for the decrease in *L. chinensis*’s ability to fix carbon through N.

### 3.5. Leaf Anatomical Structure of L. chinensis

**Figure 6:**
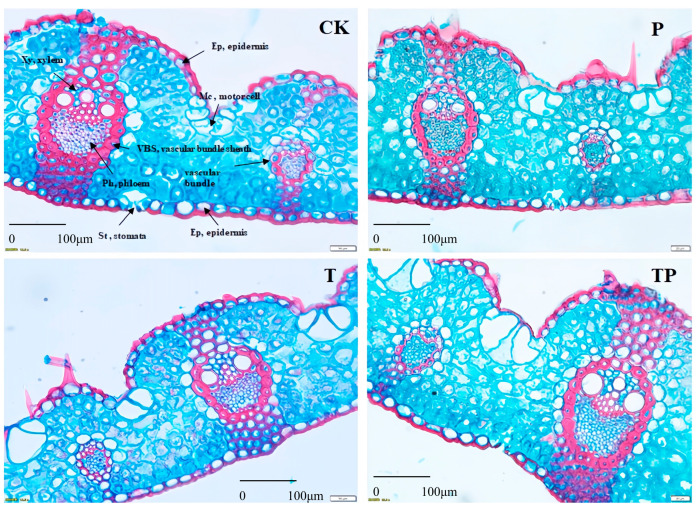
Effects of warming and precipitation addition on morphological–anatomical traits of *L. chinensis*. C: control, without warming and precipitation addition; T: warming; P: precipitation addition; TP: warming plus precipitation addition.

After observing the leaf cross-section of *L. chinensis*, it was found that the lignification degree of *L. chinensis* leaves was reduced under P as compared to the control. Significant changes were observed in the size and quantity of motor cells, the number of vascular bundles, and the number of intervals between large and small vascular bundles in the selected *L. chinensis* leaves after decadal warming and precipitation addition. The number of motor cells in the leaves of *L. chinensis* was generally 4–5; however, this number range varied (2–8) based on decade warming and precipitation addition (Table 1, Figure 6).

### 3.6. Relationship Between Photosynthetic Efficiency, Ability to Obtain Light, Resource Utilization Efficiency, Leaf Anatomical Structure, and Plant Height

**Figure 7:**
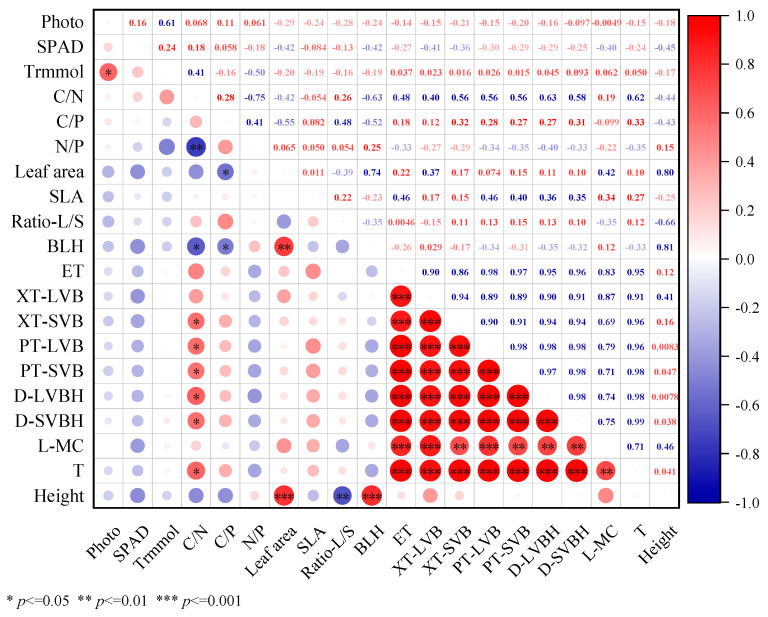
Effects of warming and precipitation addition on the height of *L. chinensis* via photosynthetic efficiency, ability to obtain light, resource utilization efficiency, and leaf anatomical structure. Photo: photosynthetic rate; SPAD: chlorophyll content of leaf; Trmmol: transpiration rate, SLA: specific leaf area, BLH: bottom leaf height, ET: epidermal thickness, XT-LVB: xylem thickness of large vascular bundle, XT-SVB: xylem thickness of small vascular bundle, PT-LVB: phloem thickness of large vascular bundle, PT-SVB: phloem thickness of small vascular bundle, D-LVBH: diameter of large vascular bundle sheath, D-SVBH: diameter of small vascular bundle sheath, L-MC: length of motor cell, T: leaf thickness. * significant at *p* < 0.05; ** significant at *p* < 0.01; *** significant at *p* < 0.001.

**Figure 8:**
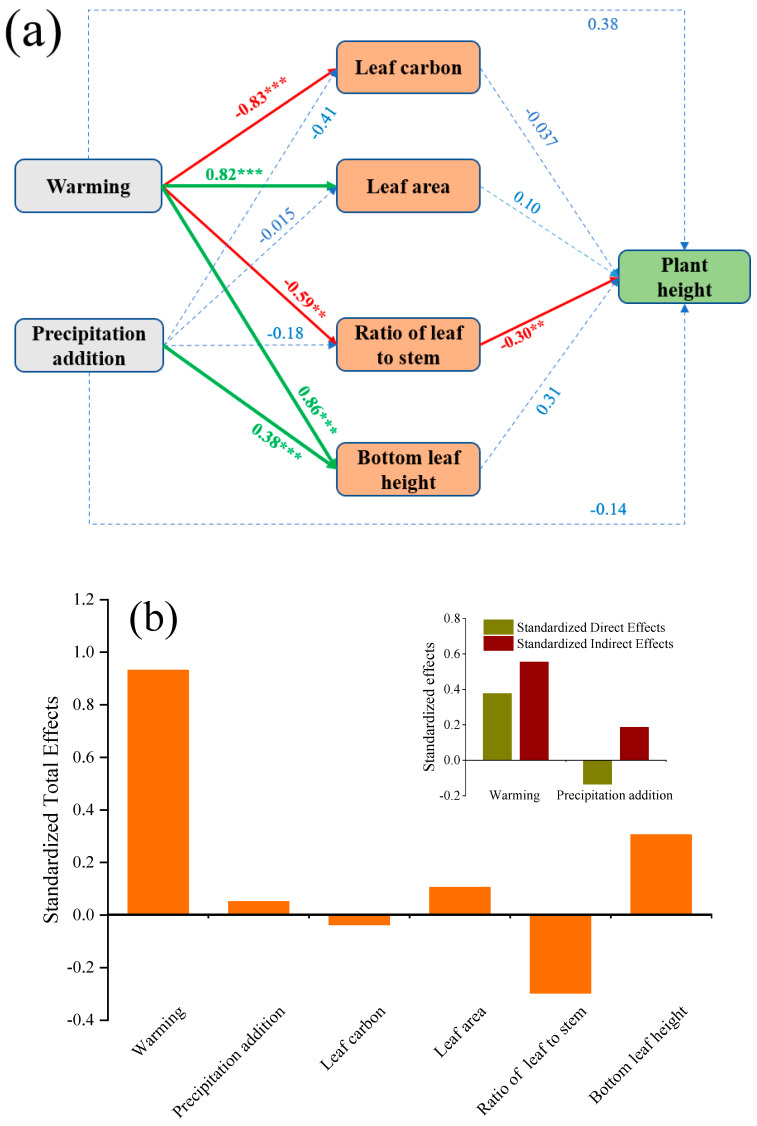
Structural equation model regarding effects of warming and precipitation addition on the height of *L. chinensis* via photosynthetic efficiency, ability to obtain light, resource utilization efficiency, and leaf anatomical structure. χ2 = 8.668; *p* = 0.653, RMSEA = 0.000. ** significant at *p* < 0.01; *** significant at *p* < 0.001. (**a**) The red line represents negative effects; the green line represents positive effects; and the blue dashed line represents insignificant effects. (**b**) The effects (values) of different factors on the height of *L. chinensis*; the small graph in the upper-right corner represents the direct and indirect effects of temperature and water addition on the height of *L. chinensis*.

In this study, the height of *L. chinensis* was significantly positively correlated with LA and bottom leaf height but significantly negatively correlated with leaf carbon content and the leaf-to-stem ratio (Figure 7). Considering the results of the structural equation model, we observed that warming conditions promoted the height of *L. chinensis* more significantly (Figure 8). The effect of warming on the height of *L. chinensis* was much greater than the effect of precipitation addition on it. Among the traits of *L. chinensis*, leaf area and bottom leaf height had a positive effect on the plant height, and an increase in leaf area and bottom leaf height was beneficial for *L. chinensis* to obtain more light (Figure 8).

## 4. Discussion

The physiological and ecological characteristics of plant species determine their position and role in a larger plant community. Previous studies have shown that *L. chinensis* adapts to different environmental conditions through a selection strategy. Several *L. chinensis* traits related to resource acquisition and environmental adaptation, such as plant height, SLA, and leaf nitrogen content, have been previously recorded as responses to changes in climate conditions [21,22,23].

Plants reduce water loss by reducing their growth to adapt to increases in water stress [25]. Furthermore, droughts lead to increased soil moisture evaporation, reduce plants’ stomatal conductance, and prevent water transpiration, thereby reducing plants’ photosynthetic rate (for adaptation to drought conditions) [33]. High temperature and drought result in a significant decrease in the Pn of *L. chinensis*, while precipitation increases significantly increase Pn. Moreover, chlorophyll content (Chl) is another important physiological indicator that reflects the photosynthetic potential of plants [20]. δ^13^C is also an important trait that reflects plants’ water use efficiency. Plants often exhibit higher δ^13^C in drought environments [34], a phenomenon that is related to drought-induced stomatal closure [28,35]. Current consensus indicates that drought stress typically constrains photosynthetic rate (Pn) through stomatal limitation-mediated reductions in transpiration [34]. However, our experimental findings reveal that warming significantly enhanced Pn in Leymus chinensis, a response potentially linked to drought adaptation mechanisms. This enhancement correlates with improved water use efficiency (WUE), as evidenced by δ^13^C signatures, aligning with the physiological trade-off framework proposed by Chaves et al. [33] wherein plants optimize carbon assimilation and hydraulic safety through dynamic stomatal regulation. Contrary to reports of synergistic suppression of Pn under combined high-temperature and drought conditions [25], our isolated warming treatment elicited stimulatory effects. This discrepancy underscores the non-linear nature of climate factor interactions, suggesting the following: single-factor responses may not predict outcomes under compound stressors, thermal and hydraulic effects exhibit context-dependent dominance, and species-specific acclimation strategies modulate stress responses. These results emphasize the critical need for factorial experimental designs to disentangle individual versus interactive climate impacts. Future investigations should prioritize mechanistic analyses of stomatal behavior, non-stomatal limitations, and biochemical plasticity under multi-stressor regimes.

Leaf nitrogen content decreases with increasing precipitation [23]. Moreover, the nitrogen content in leaves has shown a decreasing trend based on increases in a region’s annual average temperature [36]. Simulations of warming conditions using the OTC test have shown that warming significantly reduces leaf nitrogen content [37,38,39]. This is because warming promotes plant growth, while the increase in biomass dilutes nitrogen content and offsets the effects of nitrogen mineralization as well as increased plant uptake [38]. The biochemical effects of enzymes decrease at low temperatures, and plants compensate for this decrease by increasing their leaf nitrogen content [40] to offset the negative effects of low temperatures [36]. However, other studies have suggested that warming either increases the nitrogen content of leaves [36] or has no significant effect on it [41,42]. The change in a region’s leaf carbon content reflects the carbon investment of the leaves [43]; the higher the leaf carbon content, the more conservative the concomitant investment strategy. Warming significantly reduces leaf carbon content [37,41]. Generally, the carbon-to-nitrogen ratio (C/N) is used to evaluate the long-term nitrogen utilization efficiency of plants; the impact of warming on C/N varies depending on plant type and climate [44]. Warming reduces the C/N of plants’ leaves by increasing the production capacity, biological activity, and nutrient absorption of affected plants [45], thus indicating that plants may improve nitrogen utilization efficiency to adapt to a warmer future [37]. The carbon-to-phosphorus ratio (C/P) represents the ability of plants to assimilate carbon per unit of nutrient content, reflecting their nutrient utilization efficiency. Leaf phosphorus content decreases and the nitrogen-to-phosphorus ratio (N/P) increases with increases in average annual temperature on a global scale [40,44]. On the other hand, N/P tends to increase due to decreases in annual average temperature at the regional and community levels [36,46]. Previously, a study found that warming significantly increased the N/P of *L. chinensis* in a temperate meadow in northern China [47]. Most simulated warming experiments (e.g., open-top chamber studies) demonstrate reduced leaf nitrogen content, primarily attributable to biomass dilution effects [39] and temporal mismatches between plant demand and nitrogen mineralization rates [37]. Contrastingly, long-term observational data from Reich et al. (2004) revealed positive correlations between temperature and foliar nitrogen concentration [36], potentially reflecting persistent low-temperature compensation mechanisms—a physiological adaptation in which plants maintain enzymatic activity through nitrogen accumulation during short-term warming episodes. Our findings demonstrate that combined warming and precipitation modifications significantly enhanced nitrogen content, suggesting synergistic interactions between accelerated nitrogen mineralization processes and improved plant nutrient uptake capacity within this ecosystem. A consensus emerges across studies regarding warming-induced reductions in leaf carbon content, indicative of strategic shifts in plant resource allocation from carbon-conservative storage patterns toward growth-oriented metabolic investments. The observed decline in C/N ratios implies enhanced nitrogen use efficiency [44], though phosphorus dynamics warrant careful consideration. The stability of the C/P and N/P ratios in our study system may reflect regionally elevated soil phosphorus availability [47], potentially overriding phosphorus-mediated metabolic regulation observed in nutrient-limited ecosystems. This phosphorus buffering effect highlights the critical importance of site-specific biogeochemical contexts when extrapolating warming impacts on plant stoichiometry.

Environmental changes lead to substantial alterations in leaf traits; the thickness of plant leaves affects their water supply and storage capacity [48]. An increase in precipitation causes the leaves of grassland plants to become larger in areas with low to moderate precipitation around the world [49]. However, some studies have also shown that precipitation increases have had no significant effect on the SLA of the dominant species (*L. chinensis*) in the Inner Mongolia grasslands. Some scholars have observed that warming reduces the leaf thickness of *Kobresia pygmaea* [37]; this occurs due to the reduction in the leaf palisade cell area and the thinning of this species’ mesophyll tissue under high temperatures [50]. Additionally, it is also known that leaf thickness may decrease due to increases in LA [43,48]. The increase in leaf thickness has been deemed consistent with the enhancement of photosynthetic capacity per unit of LA [51]. In the current study, warming caused the leaves of *L. chinensis* to reach higher above the ground level, which also allowed these leaves to receive more light at such positions. Warming conditions led to increases in LA but did not engender decreases in leaf thickness. On the other hand, precipitation addition (TP and P) significantly reduced leaf thickness but did not change LA. The above findings indicate that the selected species’ leaf adaptation morphology varies based on specific (warming or precipitation addition) conditions. While leaf area (LA) exhibited significant increases, leaf thickness remained stable—contrasting with the negative LA-thickness relationship reported in Kobresia species under warming [37]. This apparent divergence may reflect compensatory physiological adaptations in *Leymus chinensis*, potentially mediated by enhanced photosynthetic capacity per unit leaf area through optimized mesophyll cell arrangement [51], thereby reducing selective pressure for thickness reduction. Alternatively, observed patterns of stable LA coupled with reduced leaf thickness align with the “thin-leaf rapid expansion” strategy [43], though these findings partially contradict global meta-analyses documenting preferential leaf enlargement in low–medium precipitation regimes [49]. Such discrepancies suggest the existence of species-specific thresholds in morphological responses to environmental drivers, possibly modulated by evolutionary adaptations to regional hydroclimate conditions and intrinsic growth constraints. This highlights the necessity of considering the phylogenetic context when generalizing plant functional responses to climatic changes.

The position and development trend of dominant plant species in a community greatly affect the plant community’s stability and species diversity [6,52,53,54]. They are the ‘drivers’ or decision-makers of ecosystem processes and have a profound impact on them [6,10,55]. Dominant species have a greater competitive advantage over other species in resisting environmental interference, which renders them more sensitive to climate change [11,12]. The enhancement of the position of a community’s dominant plants creates strong competitive pressure on its subordinate species [56,57]. On the contrary, other studies have shown that warming significantly promotes the growth of coexisting species and reduces inter-species competition in semi-arid grasslands [58]. Warming has led to an increase in the population density and productivity of *L. chinensis* in the Eurasian temperate grasslands [19,59]. However, warming has also led to a decrease in the number of dominant species in the temperate grasslands of northern China [60]. To be precise, in arid and semi-arid regions, precipitation is the main factor that affects plant growth. Drought has further led to increases in the biomass of the dominant species (*L. chinensis*) in the Songnen grasslands of northern China [59]. Precipitation increase has also promoted the growth of competing species, resulting in a decrease in the dominant position of *L. chinensis* [60]. However, additional precipitation has significantly enhanced the biomass accumulation of *L. chinensis*, whereas drought caused by decreased precipitation has led to dramatic reductions in biomass [19]. This study demonstrates that *Leymus chinensis* compresses its ecological niche through light competition, reducing community diversity—a pattern supporting the “dominant species-driven hypothesis” [10] and aligning with observations [59]. However, in certain temperate grasslands, warming-induced declines in dominant species’ dominance [61] may reflect an “environmental filtering-competition trade-off” [56], where resource limitations (e.g., water scarcity) elevate competition costs beyond adaptive benefits. The paradoxical drought-mediated promotion of *L. chinensis* [59] and hybrid grasses [62] likely arises from drought gradient effects: moderate drought enhances dominant species via competitor screening, whereas extreme drought thresholds trigger ecosystem destabilization [63]. This intensity-dependent response highlights nonlinear ecological outcomes shaped by stress magnitude and temporal persistence, underscoring the critical role of disturbance regimes in mediating competition dynamics and community assembly.

Under climate change pressures, *Leymus chinensis* dominance in Inner Mongolia’s typical grasslands exhibits pronounced height augmentation alongside reduced community diversity. Warming trends and altered precipitation regimes favor the expansion of this C3 rhizomatous grass, while disadvantaging drought- and cold-tolerant specialists. To preserve grassland ecological integrity, we propose the following: (1) implementation of adaptive rotational grazing regimes synchronized with seasonal biomass fluctuations to mitigate selective overgrazing of *L. chinensis* while facilitating subordinate species recovery; (2) ecological niche complementation through targeted reseeding of degraded patches coupled with strategic reintroduction of native forbs; and (3) policy frameworks prioritizing functional diversity metrics over purely productivity-based assessments, as multispecies assemblages demonstrate greater resilience buffering against climatic extremes. Critically, these interventions must account for non-linear species responses to interacting stressors—while moderate warming enhances *L. chinensis* competitiveness, threshold exceedance in aridity indices could precipitate the systemic collapse of its dominance hierarchy. This management paradigm emphasizes dynamic equilibrium maintenance rather than static conservation targets, acknowledging grassland ecosystems as climate-responsive metacommunities.

## 5. Conclusions

*L. chinensis* reached significantly greater heights under warming conditions. Warming also increased the LA of *L. chinensis* but decreased its leaf carbon content significantly. Moreover, warming and precipitation addition regulated the height of *L. chinensis* by affecting LA, leaf–stem ratio, distance of the bottom leaf from ground level, and leaf morphology–anatomy traits. Our results provide reasonable predictions regarding the succession direction of Inner Mongolia’s *L. chinensis* steppe and support the protection of its temperate steppe in light of climate change in the future. We provide evidence for the argument that a warmer and wetter future will be beneficial to the growth of *L. chinensis* in this steppe. Although this may be beneficial for the utilization of animal husbandry, it will not be conducive to the diversity of community species in the steppe. Therefore, future studies on the broader issue should pay more attention to the changes in the number of species in grassland communities, especially those with lower dominance levels within such communities.

## Figures and Tables

**Table 1:** Quantitative morphological–anatomical traits of ***L. chinensis***. C: control, without warming and precipitation addition; T: warming; P: precipitation addition; TP: warming plus precipitation addition.

	CK	T	TP	P
Epidermal thickness (µm)	20.88 ± 0.68 a	22.34 ± 0.71 a	12.34 ± 0.28 b	12.17 ± 0.31 b
Xylem thickness of large vascular bundle (µm)	75.60 ± 5.22 a	85.57 ± 7.56 a	50.17 ± 2.03 b	35.24 ± 1.97 c
Xylem thickness of small vascular bundle (µm)	34.75 ± 1.55 a	32.74 ± 1.89 a	20.22 ± 0.91 b	13.82 ± 0.83 c
Phloem thickness of large vascular bundle (µm)	75.64 ± 1.68 a	74.61 ± 2.36 a	42.66 ± 0.45 b	44.57 ± 0.91 b
Phloem thickness of small vascular bundle (µm)	60.56 ± 1.24 a	56.39 ± 1.86 a	36.45 ± 0.67 b	33.25 ± 0.94 c
Diameter of large vascular bundle sheath (µm)	27.50 ± 1.05 a	25.57 ± 0.92 a	12.06 ± 0.37 b	11.18 ± 0.24 b
Diameter of small vascular bundle sheath (µm)	16.97 ± 0.60 a	15.85 ± 0.48 a	8.29 ± 0.23 b	7.71 ± 0.18 b
Length of motor cell (µm)	59.12 ± 1.40 b	90.33 ± 2.44 a	47.07 ± 1.13 c	47.96 ± 1.35 c
Leaf thickness (µm)	339.86 ± 17.71 a	316.05 ± 12.02 a	199.34 ± 6.62 b	177.59 ± 5.35 c

Note: Different letters indicate significant differences in each index between different treatments.

## Data Availability

Data are contained within the article.
